# Exploration of Cervical Cancer Image Processing and Detection Based on U-RCNNs

**DOI:** 10.2174/0115734056333197241211162651

**Published:** 2025-01-02

**Authors:** Cheng Cheng, Yi Yang, Youshan Qu

**Affiliations:** 1 Changchun University of Science and Technology,7089 Weixing Road, Changchun City, Jilin Province, China; 2 Department of Radiotherapy, The Second Norman Bethune Hospital of Jilin University,No.218 Ziqiang Street, Nanguan District, Changchun City, Jilin, China; 3 Xi'an Institute of Optics and Precision Mechanics of CAS, No.17, Information Avenue, New Industrial Park, Gaoxin District, Xi 'an, China

**Keywords:** U-RCNNS, Cervical cancer, Image processing, Deep learning, Early detection, Segmentation, Object detection, Medical imaging, Artificial intelligence

## Abstract

**Background:**

Cervical cancer is a prevalent malignancy among women, often asymptomatic in early stages, complicating detection.

**Objective:**

This study aims to investigate innovative techniques for early cervical cancer detection using a novel U-RCNNS model.

**Methods:**

Cervical epithelial cell images stained with hematoxylin and eosin (HE) were analyzed using the U-RCNNS model, which integrates U-Net for segmentation and R-CNN for object detection, incorporating dilated convolution techniques.

**Results:**

The U-RCNNS model significantly improved the accuracy of detecting and segmenting cervical cancer cells, with the enhanced Mask R-CNN showing notable advancements over the baseline model.

**Conclusion:**

The U-RCNNS model presents a promising solution for early cervical cancer detection, offering improved accuracy compared to traditional methods and highlighting its potential for clinical application in early diagnosis.

## 1 INTRODUCTION

In recent years, the advent of deep learning technology has reshaped perspectives on early cervical cancer detection. Deep learning techniques autonomously acquire features within images, leading to significant strides in the detection and localization of cervical cancer cells. This study investigates methods for cervical cancer image processing and detection using the U-RCNNS model. Employing cervical epithelial cells stained with hematoxylin and eosin (HE) as the research material, image processing, and detection were conducted through the U-RCNNS model. U-RCNNS, an integration of U-net and RCNN, utilizes U-net for image segmentation and RCNN for object detection, enabling precise detection and localization of cervical cancer cells. This paper closely examines English literature related to deep learning-based segmentation of cervical cell images and associated medical diagnostics. The reviewed studies aim to improve the precision and automation of cervical cell image segmentation using deep learning, thereby providing effective support for early diagnosis and prevention of cervical cancer. The review encompasses deep learning-based segmentation and diagnosis of cervical cell images, as well as closely related medical literature. These studies utilize deep learning techniques to enhance the automatic diagnostic accuracy for cervical cancer patients, automate the registration of image sequences, and improve the design of intelligent models, thereby offering robust support for the early detection and treatment of cervical cancer.

Unsupervised deep learning-based uterine cervical sequence image registration methods were discussed by Guo *et al*. [1]. These methods employ deep learning for the automated registration of cervical sequence images, laying the foundation for subsequent analysis and medical image diagnostics. This research provides potential solutions for the early detection of cervical cancer. The optimal application of the deep learning Inception model in cervical cancer diagnosis was investigated by AbuKhalil *et al*. [2]. Through deep learning, an optimized Inception model was designed to enhance the accuracy of cervical cancer diagnosis, offering a novel direction and approach for automated cervical cancer diagnosis.

Anupama *et al*. [3] focused on applying intelligent classification models to biomedical cervical smear images in conjunction with the Internet of Things (IoT) environment. They designed an intelligent classification model that, when combined with IoT technology, achieved intelligent classification of cervical smear images. This study holds promising potential for application in cervical cancer diagnosis.

Waly *et al*. [4] concentrated on designing an optimal deep convolutional neural network model for cervical cancer diagnosis. By optimizing deep learning models, the efficiency and accuracy of cervical cancer diagnostic models can be enhanced, providing new insights for further research in the field of cervical cancer diagnosis.

These studies illustrate the application of deep learning techniques in cervical cancer diagnosis, image registration, and the design of intelligent classification models. Innovative methods and technologies for early detection, diagnosis, and treatment of cervical cancer are available, with the continuous development and refinement of deep learning promising further breakthroughs and advancements in medical image processing [5].

## 2 MATERIALS AND METHODS

### 2.1 Technical Roadmap based on Cervical Cell Images

#### 2.1.1 Pathological Analysis of Cervical Cells

Pathological analysis of cervical cells plays a crucial role in the prevention, diagnosis, and treatment of cervical cancer. Two types of normal cervical cells exist squamous epithelial cells and columnar epithelial cells. Abnormal epithelial cells and those displaying signs of carcinogenesis serve as precursors and markers of cervical cancer development.

Within normal cervical cells, squamous epithelial cells are partially distributed in the cervical os and part of the cervical canal, arranged in layers, typically comprising approximately 20–30 layers. Squamous epithelial cells primarily function to prevent external infections and damage and are capable of self-renewal through division. Columnar epithelial cells are present in most areas of the cervical canal, arranged in columnar or cylindrical shapes, with a height of approximately 10–15 cells. The main roles of columnar epithelial cells include secreting mucus, lubricating the vagina, and protecting the cervix from harm [6]. Additionally, cervical epithelial cells include other types, such as squamous-columnar transitional epithelium and vesicular epithelium.

Abnormal epithelial cells within normal cervical cells refer to those exhibiting cytological and/or histological abnormalities, often considered precursors of carcinogenesis. For example, cervical intraepithelial neoplasia (CIN) is a common type of abnormal epithelial cell characterized by varying degrees of cellular atypia and proliferation without disruption of the basal layer basement membrane. CIN is classified into three levels: CIN1, CIN2, and CIN3, with CIN3 representing the highest level of abnormal epithelial cells, most closely related to carcinogenic changes.

Carcinogenic cells are cancerous cells appearing within epithelial cells, exhibiting atypical characteristics and abnormal growth fundamentally different from that of normal cells. Cervical cancer typically originates from the transformation zone of the cervical epithelium, which is the junction between the squamous and columnar epithelium [7].

HE staining, a widely employed tissue staining technique, stains cell nuclei purple and cytoplasm pink, elucidating the morphology, structure, and distribution of cells. This study aims to investigate methods for cervical cancer image processing and detection based on HE-stained exfoliated cervical cells using U-RCNNS. Fig. (**1**) presents an illustrative image of HE-stained cervical exfoliated cells, showing the cellular characteristics. U-RCNNS, a convolutional neural network based on deep learning, efficiently processes and recognizes images. H&E-stained exfoliated cervical cells encapsulate morphological details concerning cervical cells and staining information regarding cell nuclei [8].

The method for cervical cancer image processing and detection using U-RCNNS entails HE staining of exfoliated cervical cells, followed by analysis and processing employing U-RCNNS. This process extracts morphological features of cervical cells and staining information from cell nuclei, facilitating the identification and detection of cervical cancer.

### 2.2 Enhanced Techniques based on Sample Data Sets

Addressing the common issue of Object/Box-level imbalance in object detection, this paper introduces an augmentation technique based on a sample dataset that employs the FPN architecture.

In object detection, the commonly used method involves detecting candidate boxes, which may contain false positives (FP), representing false detections of objects. Moreover, the imbalance between positive and negative samples at the box level for certain categories can diminish model performance due to a relatively lower number of samples in the training set. Therefore, this enhancement technique aims to mitigate these issues through thorough exploration and research.

Specifically, the augmentation technique in this paper consists of several steps. Firstly, an analysis of the training dataset identifies the issue of Box-level imbalance. Secondly, adjusting the ratio of positive to negative samples in the dataset alleviates this problem. Subsequently, the utilization of the feature pyramid network (FPN) architecture enhances the samples to further improve the model's performance. Finally, through an analysis of the experimental results, the effectiveness and practicality of the proposed augmentation technique are validated [9].

In contrast to traditional data augmentation methods, the technique presented in this paper effectively addresses object/box-level imbalance issues. The FPN architecture is employed for enhancement, retaining spatial information within images more effectively. The method demonstrated in this paper exhibits superior performance in terms of detection accuracy, precision, recall, and other aspects, as confirmed by the analysis of the experimental results, validating its effectiveness and practicality. As shown in Fig. (**2**).

### 2.3 Morphological Estimation Based on Rotated Ellipses

Morphological estimation based on rotated ellipses is a geometric image processing method employed for tasks such as object detection, tracking, and recognition. Its fundamental principle involves fitting the target area into a rotated ellipse and subsequently utilizing the geometric characteristics of the ellipse for object detection and recognition.

In the process of morphological estimation, the initial step entails defining the standard equation of the ellipse. For a rotated ellipse on a plane, the standard equation is:


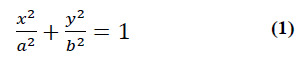


Where (x, y) represents the coordinates of the center of the ellipse, and 'a' and 'b' denotes the major and minor axes of the ellipse, respectively.

Subsequently, considering the specific characteristics of the actual target area, the standard ellipse requires translation and rotation to align with the real shape of the target. After translation and rotation, the coordinate formula for the ellipse is as follows:


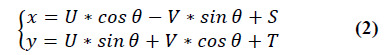


Where (x, y) represents the initial coordinates of the ellipse, and θ represents the rotation angle.

Estimating morphology using an ellipse on the target area enables the extraction of geometric characteristics, including the aspect ratio and area. This facilitates tasks such as detection, tracking, and recognition. Furthermore, utilizing the geometric features of the ellipse allows for shape analysis and classification of the target, expanding the range of applications.

Let UOV denote the Cartesian coordinate system of the right-handed spiral plane, acquired after translation:


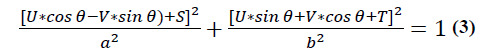


The representation of the ellipse in the UoV coordinate system can be expressed in the XY coordinate system as follows:


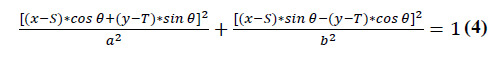


The specific parametric form of the coordinates of point P after translation and rotation in the XY system can be represented as follows:


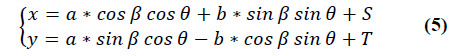


### 2.4 Label Fusion Strategy

Label fusion involves consolidating labels obtained from various models or methods to enhance object detection or segmentation accuracy. In tasks related to object detection and segmentation, diverse models may annotate the same object differently, necessitating label fusion for improved detection accuracy.

Among these methods, Mask R-CNN segmentation and U-Net nucleus segmentation are commonly employed. Mask R-CNN is an instance segmentation model within the R-CNN framework, capable of simultaneously detecting and segmenting multiple instance targets. U-Net nucleus segmentation, on the other hand, is a pixel-level segmentation model based on convolutional neural networks, utilizing its U-shaped architecture for effective handling of detailed information.

In the label fusion process, the watershed cytoplasmic segmentation method can be applied for pixel-level annotation. This method segments the image, assigning each pixel to regions corresponding to different objects. Analyzing pixel features in these regions enhances the accuracy of labeling results. Simultaneously, it is crucial to consider differences between labels obtained from different models or methods at both the pixel and instance levels during the label fusion process. Therefore, methods such as weighted averaging or voting can be employed for label fusion, ensuring more accurate outcomes. As shown in Fig. (**3**).

## 3 EXPERIMENT ON CERVICAL CELL IMAGE PROCESSING BASED ON U-NET

### 3.1 U-Net Architecture

U-Net is a deep-learning neural network architecture that was designed for image segmentation tasks. It was initially introduced by Ronneberger *et al*. in 2015, and the name originates from the shape of its network structure, resembling a 'U'. It comprises a contracting path (encoder) and an expansive path (decoder). U-Net architecture is based on the fully convolutional network (FCN) framework, notable for its robustness to small-sized objects and background noise [10].

The U-Net algorithm is fundamentally comprised of two components: the encoder (contracting path) and the decoder (expansive path). In the encoder, U-Net employs a down-sampling network structure consisting of convolutional and pooling layers to extract image features. The decoder utilizes up-sampling and convolution operations to progressively expand feature maps. Moreover, skip connections are employed to connect low-level feature maps from the encoder with high-level feature maps from the decoder, preserving more semantic information. A noteworthy characteristic of U-Net is the inclusion of a convolutional layer in the final network layer, producing pixel-level segmentation results that match the original image size.

The contracting path of U-Net is composed of iteratively applied convolutional blocks, with each block containing two convolutional layers and one max-pooling layer. This architecture systematically reduces the size and channels of feature maps while elevating their abstraction levels [11]. In the expansive path, U-Net employs up-sampling and convolutional layers to progressively regain the size and channels of feature maps. The inclusion of skip connections combinates feature maps from both the encoder and decoder, facilitating the network in learning features of varied scales and thereby improving segmentation performance. As shown in Fig. (**4**).

Through its structured contracting and expansive paths, U-Net achieves seamless integration of image features and uses global information, resulting in exceptional performance in the domain of image segmentation. The U-Net architecture diagram is presented below [12].

### 3.2 Segmentation Method based on U-Net

In U-Net training, the standard practice involves using pixel-level cross-entropy loss functions, which do not consider the relative positional relationships between pixels. To overcome this limitation, researchers introduced a weighted loss method. This method assigns different weight values to pixels with diverse labels in training samples, enabling the network to concentrate more on challenging-to-classify pixels. Additionally, adopting multitask learning simultaneously takes into account pixel-level classification and bounding box prediction, thereby enhancing the network's accuracy [13]. For cytoplasm segmentation, common techniques include using watershed algorithms or connectivity algorithms for boundary detection. However, these methods have limitations, particularly in distinguishing complex boundaries between multiple cells. Fig. (**5**) displays the cervical cell segmentation results based on U-Net. To address this challenge, researchers proposed a segmentation method based on cytoplasm segmentation contours. This method employs the U-Net to predict the classification probability for each pixel. Subsequently, based on a threshold, cytoplasm segmentation is performed to extract accurate cytoplasmic segmentation contours. In image segmentation tasks, overlapping regions between objects can lead to errors in prediction results [14]. To mitigate severe overlap issues, researchers introduced an original b-based method. Fig. (**6**) illustrates the extraction of cervical cell cytoplasm segmentation contours using U-Net. This approach uses U-Net to predict the classification probability for each pixel. The overlap between adjacent pixels is then optimized using the Original b algorithm, resulting in accurate segmentation outcomes.

## 4 RESULTS AND DISCUSSION

The enhanced Mask R-CNN is a neural network algorithm employed for object detection and segmentation, prominently utilized in medical image analysis. In this experiment, 100 cervical cell sample images from the ISBI2014 dataset were utilized to investigate the impact of dilated convolutions in the improved Mask R-CNN [15].

The experimental process primarily involves the following steps:

### 4.1 Data Preparation

We randomly selected 100 cervical cell sample images from the ISBI2014 dataset and performed data preprocessing and augmentation, including operations such as rotation, scaling, and cropping, among others, to acquire a more extensive set of sample data.

### 4.2 Network Architecture Construction

Construct the network architecture based on the improved Mask R-CNN algorithm. The original algorithm is enhanced by incorporating dilated convolutions to expand the network's receptive fields and efficacy, ultimately improving the accuracy of target detection and segmentation.

### 4.3 Parameter Settings

Configure network parameters, including the learning rate, number of training epochs, and batch size. To mitigate model overfitting, implement L2 regularization and dropout techniques.

### 4.4 Model Training

Utilize preprocessed and increased datasets for model training. Adjust and optimize the network based on variations in the training loss function to attain optimal model performance [16].

### 4.5 Model Evaluation

Evaluate the model using the test set data from the ISBI2014 dataset. Consider metrics such as accuracy, recall, and F1 score for both target detection and segmentation. As shown in Fig. (**7**).

The main parameters include:

(1) Dilated Convolution Parameters: Dilation rate set to 2, with a dilated convolution kernel size of 3 × 3.

(2) Network Parameters: Initial learning rate of 0.001, 50 training epochs, batch size of 16, L2 regularization parameter of 0.01, and dropout parameter of 0.5.

(3) Data Augmentation Parameters: The rotation angle ranged between 10 and 90 degrees, the scaling ratio ranged between 0.8 and 1.2, and the image cropping size was set at 256 × 256 pixels.

(4) Training Loss Function: Optimization by combining the cross-entropy loss function and mean squared error loss function, achieved by setting equal weights of 0.5 for both loss functions.

## CONCLUSION

The optimization of Mask R-CNN through the implementation of dilated convolutions yielded encouraging results in this study. This innovative approach holds substantial promise for enhancing the diagnosis and treatment of various medical conditions, particularly in the realm of medical image analysis. Specifically, in the context of cervical cancer detection, the improved performance of the U-RCNNS model demonstrates its potential to significantly advance early diagnostic capabilities. By refining image segmentation and detection techniques, this method may lead to more accurate and timely interventions, ultimately contributing to better patient outcomes.

## Figures and Tables

**Fig. (1):**
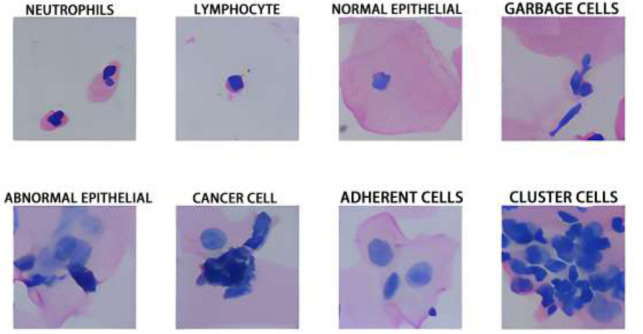
Example image of cervical exfoliated cells based on HE staining.

**Fig. (2):**
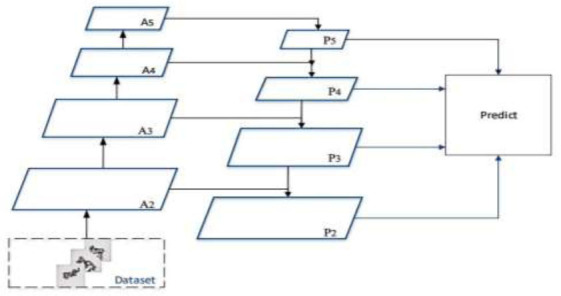
Fundamental framework of FPN.

**Fig. (3):**
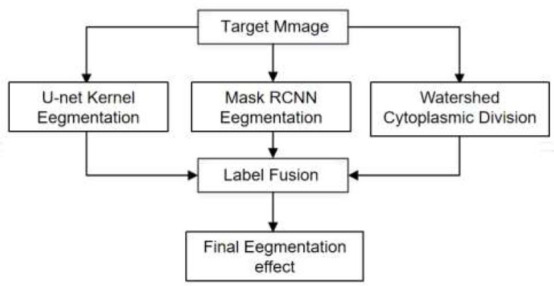
Mask RCNN segmentation flowchart.

**Fig. (4):**
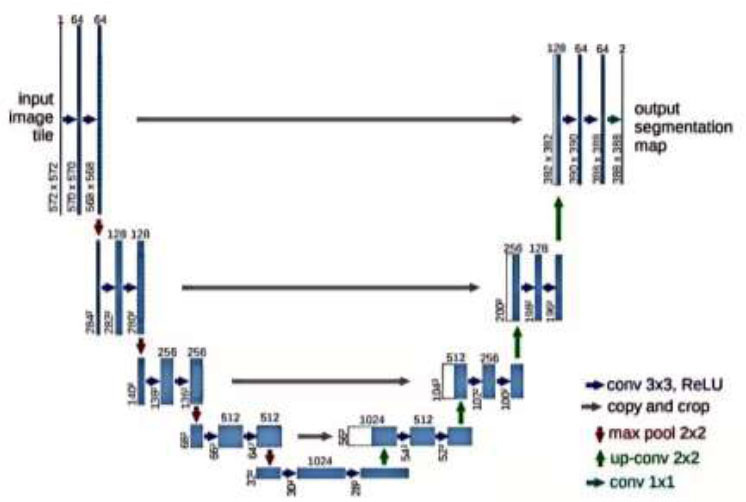
U-Net architecture diagram.

**Fig. (5):**
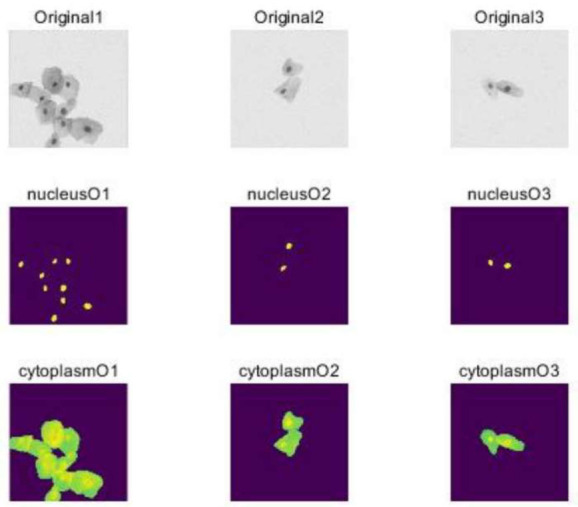
Cervical cell segmentation results based on U-Net.

**Fig. (6):**
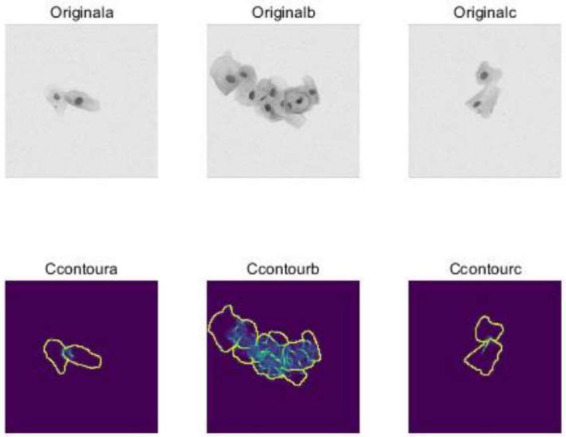
Cytoplasm segmentation contour extraction results based on U-Net.

**Fig. (7):**
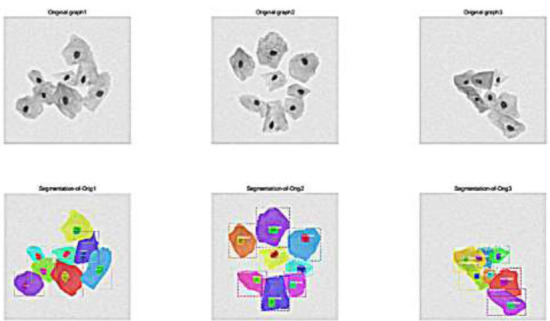
Segmentation results of cervical cells using mask R-CNN based on dilated convolutions and label fusion.

## Data Availability

All the data and supporting information is provided within the article.

## LIST OF ABBREVIATIONS

IoT: Internet of Things
CIN: Cervical Intraepithelial Neoplasia
FP: False Positives
FPN: Feature Pyramid Network
FCN: Fully Convolutional Network
